# Organocatalytic vat-ring-opening photopolymerization enables 3D printing of fully degradable polymers

**DOI:** 10.1038/s42004-023-00985-4

**Published:** 2023-08-21

**Authors:** Satoshi Honda

**Affiliations:** Graduate School of Arts and Sciences, The University of Tokyo, 3-8-1 Komaba Meguro-ku, Tokyo, 153-8902 Japan

**Keywords:** Polymers, Polymer synthesis, Design, synthesis and processing

## Abstract

Vat-polymerization 3D printing (3DP) enables the high speed printing of precise and intricate 3D models, yet it inevitably produces highly crosslinked polymers that are not easily degradable or recyclable. Here, the author highlights recent work that realizes the formation of fully degradable polymers based on organocatalytic vat-ring-opening photopolymerization 3DP.

Vat-polymerization (VP) 3D printing (3DP) has received significant attention owing to its advantages, including high printing speed, available material scope, and printing resolution. However, in contrast to fused deposition modeling (FDM)-type 3DP, which can use thermoplastic resins such as polylactide, VP-3DP inevitably produces thermosets, or highly crosslinked polymers, that are not easily degradable or recyclable. Therefore, the development of materials and processes that can address this problem is imperative for a sustainable future.

## History and challenges of vat-photopolymerization 3D printing

**Fig. 1:**
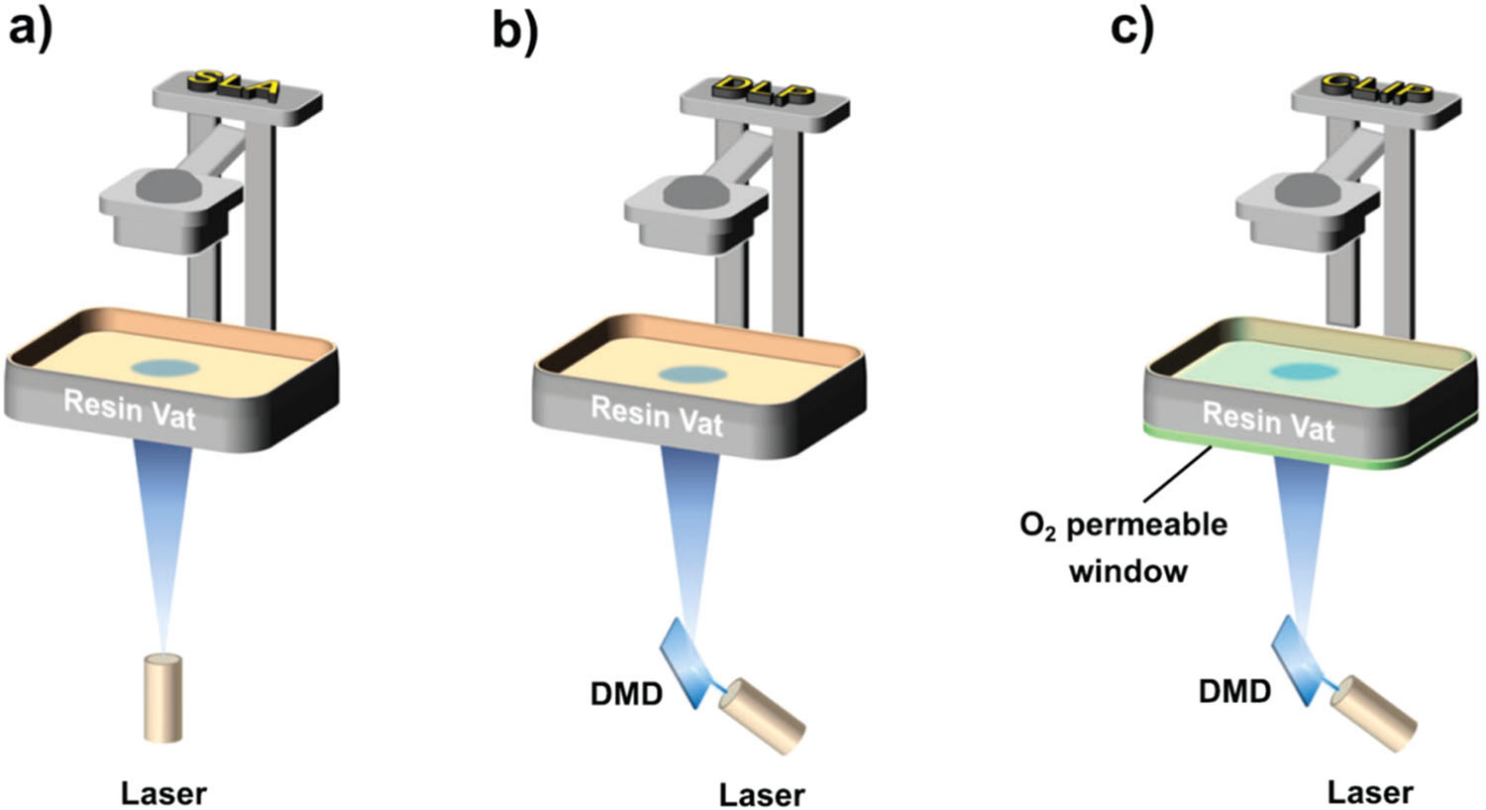
Schematic illustrations of representative vat-photopolymerization 3D printings. (**a**) Stereolithography (SLA), (**b**) digital light processing (DLP) and (**c**) continuous liquid interface production (CLIP)-type 3D printings. Copyright: Modified from ref. ^2^, used under CC BY.

Since the invention of the first rapid prototyping system by Hideo Kodama in 1981 that utilizes photopolymerization of liquid resins or monomers layer-by-layer and to fabricate 3D models consisting of stacked 2D layers^1^, additive manufacturing, commonly known as 3D printing (3DP), has impacted our daily lives. The pioneering 3DP methodology developed by Kodama is currently known as vat-photopolymerization (VP) 3DP and has received significant attention owing to advantages including high printing speed, available monomer scope, and printing resolution. In addition to commercially available stereolithography (SLA)-, digital light processing (DLP)-, and continuous liquid interface production (CLIP)-type 3D printers (Fig. 1), low-cost household liquid crystalline display (LCD)-type 3D printers basically rely on VP-3DP, and the 3DP market is expanding in combination with the progress of devices / therein^2–5^. The process of VP-3DP uses liquid-state mixtures of photoinitiators, monomers, oligomers, and crosslinkers that undergo photopolymerization. To rapidly form intricate 3D models, VP-3DP generally requires a large amount of photoinitiators and crosslinkers and thereby inevitably produces nondegradable thermosets. However, this aspect discourages material recyclability and brings limited adaptability and restricted reprocessability. To solve this problem, several recent reports have focused on dynamic covalent materials to create reversibility in 3D-printed materials^6,7^, as represented by thermoresponsive motifs that rely on the Diels–Alder reaction^8,9^.

On the other hand, the demand for polymers composed of highly degradable repeating units, such as poly(lactic acid) (PLA), polycaprolactone (PCL), and poly(trimethylene carbonate) (PTMC), to mention a few, is rapidly increasing to realize a sustainable society. Such readily degradable polymers, or polyesters and polycarbonates, have long been used in fused deposition modeling (FDM)-type 3DP, which is characterized by the extrusion of heated polymer melts through a nozzle. However, the diameter of the nozzle that can extrude polymers is 0.1–0.2 mm at the smallest, making it more difficult to achieve more precise and intricate modeling than with VP-3DP^10^. In addition, because it is necessary to scan the nozzle in the xy direction for each layer, it is much more time-consuming than DLP-, CLIP-, and LCD-type VP-3DP, which is capable of stacking one layer of light-exposed areas that are instantly cured face by face. Therefore, if easily degradable polyester and polycarbonate could be directly modeled by VP-3DP, it would be extremely innovative, and there would be no doubt that it will contribute to the realization of a sustainable society.

## History of photobase generator and vat-ring-opening photopolymerization 3D printing

**Fig. 2:**
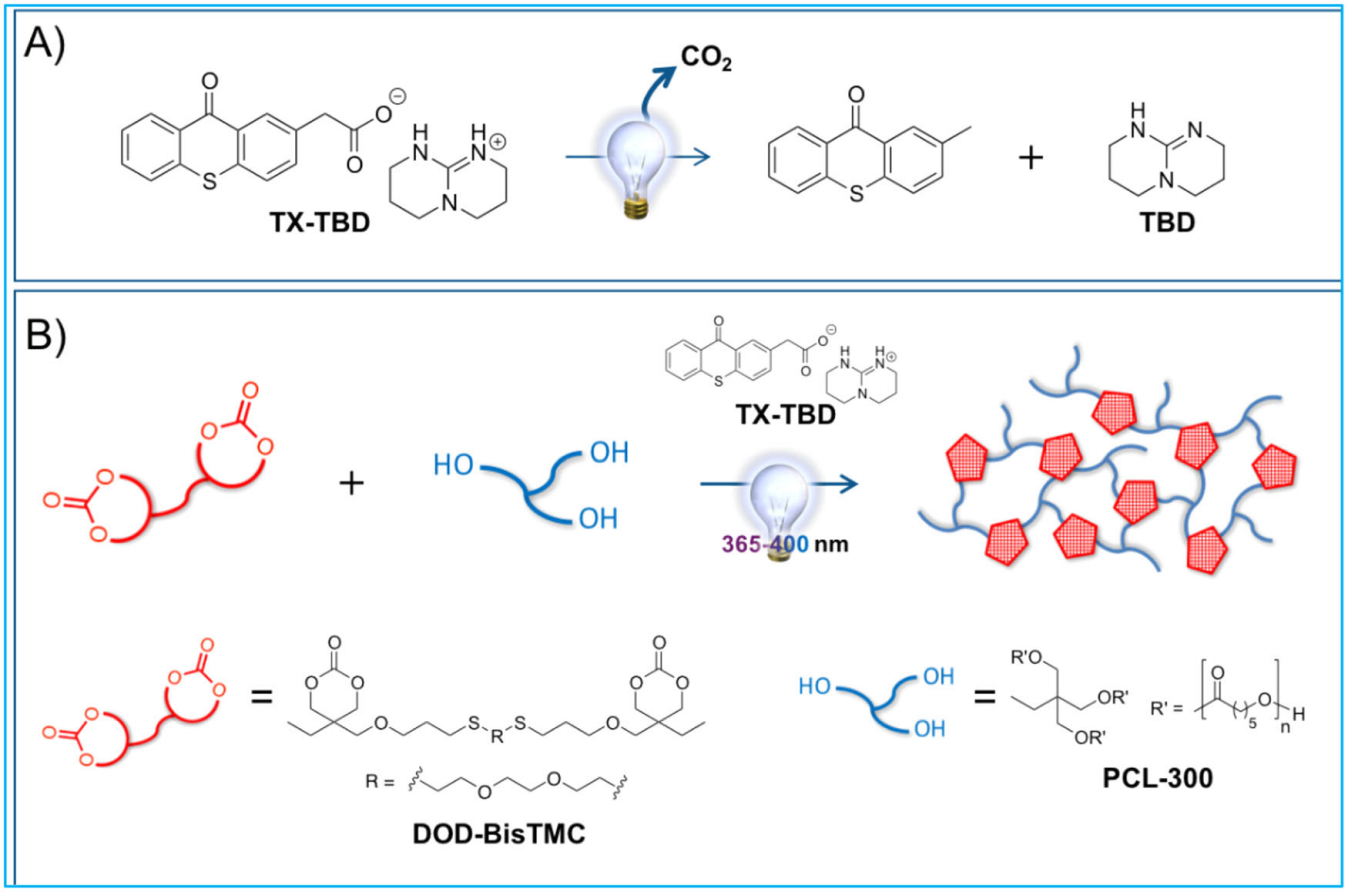
Organocatalytic ring opening polymerization enabled by a photobase generator. (**A**) The photobase generation reaction of thioxanthone acetic acid-based salt of TBD. (**B**) Vat-ring-opening photopolymerization reaction for the 3DP of fully degradable polymers. Copyright: Adapted from ref. ^11^, used with permission from Elsevier.

This idea has recently been achieved^11^ and is gradually spreading. The key is the use of a photobase generator (PBG)^12^. Conventional VP-3DP has cured resins by inducing radical or cationic polymerization using either a photoradical or a photacid generator^2^. On the other hand, PBG, which generates a base upon photostimulation, is newer than photoradical and photacid generators and was first reported in 1987^13^, 6 years after the invention of VP-3DP by Kodama^1^. With the progress of catalytic chemistry, it was also reported that organic strong bases, such as 1,5-diazabicyclo[5.4.0]undec-5-ene, 1,5-diazabicyclo[4.3.0]non-5-ene, and 1,5,7-triazabicyclo[4.4.0]dec-5-ene (TBD), exhibit extremely high catalytic activity for ring opening polymerization (ROP) of lactones and cyclic carbonates^14,15^, enabling the synthesis of polyesters and polycarbonates with controlled molecular weights and dispersities in an extremely shorter time than those of conventional polycondensation reactions. Therefore, PBGs capable of producing organic strong- or super-bases upon photoirradiation may open up new 3DP methods, which can be named, for example, as vat-ring-opening photopolymerization (VROP) 3DP. Perhaps, a significant advance in the design of PBGs has been made 20 years after the first report of PBG^13^ by the development of bicyclic guanidinium tetraphenylborate, which produces TBD upon photoirradiation, as reported by Sun et al. 2008^16^. Since this report, a number of PBGs have been reported. A ketoprofenate salt of TBD reported by Arimitsu et al. is based on the decarboxylation reaction of ketoprofen, and its quantum yield (Φ_313_) is as high as 0.75^17^. This quantum yield is four times higher than that of the tetraphenylborate salt of TBD (Φ_254_ = 0.18) reported by Sun et al.^17^, thereby becoming industrially important. In fact, the first report of the ketoprofenate salt of superbases, including 2-(9-oxoxanthen-2-yl)propionic acid 1,5,7-triazabicyclo[4.4.0]dec-5-ene (OX-TBD), was published in 2013^17^, and related PBGs were commercialized immediately after the publication. After 9 years, VROP-3DP by utilizing thioxanthone acetic acid-based salt of TBD (TX-TBD) (Fig. 2A), which is an analog of OX-TBD, was achieved by Zivic et al.^11^ While common ROP reactions were conducted in solution, they polymerized a solventless mixture of a trimethylene carbonate bis-functionalized monomer (DOD-BisTMC) and a PCL-based trifunctional polyol oligomer (PCL-300) as a crosslinker and initiator in the presence of TX-TBD upon photoirradiation (*λ* = 385 nm) (Fig. 2B). It is important that the polymerization mixture be liquid in a ‘vat’ when performing common VP-3DP. One of the important points in this study is that both/DOD-BisTMC and PCL-300 are in liquid states. However, simply mixing TX-TBD with DOD-BisTMC and PCL-300, even if they are liquid compounds, did not form a homogeneous mixture, and no photocuring reaction occurred. This means that another important point is how to homogenize a highly polar organic salt-based TX-TBD in the polymerization mixture. They found that once all of DOD-BisTMC, PCL-300, and TX-TBD were dissolved in dichloromethane and then the dichloromethane was evaporated, the resulting homogeneous polymerization mixture underwent a photocuring reaction. On the other hand, they also observed a dark polymerization process from the existence of the time at which storage modulus and loss modulus cross each other after completing irradiation and a photocuring process beyond the irradiated area. As some PBGs, including commercially available OX-TBD, show autocatalytic base amplification, which is characterized by the accelerated decomposition of the parent PBG by the generated base^18,19^, these results suggest the possibility of achieving further improvements in the design of PBG or ROP conditions for more precise 3DP of more intricate 3D models. Nevertheless, the produced material should be fully degradable, as indicated by the chemical structure (Fig. 2B), and the present methodology could, in principle, be applied to VROP-3DP of various polyesters and polycarbonates.

## Outlook

The work by Zivic et al. opened a new avenue for the VROP-3DP of fully degradable polymers based on the effective utilization of PBGs to cause polymerization of liquid bislactone crosslinkers initiated from liquid trifunctional polyol oligomers upon photoirradiation. It should be noted that this method can, in principle, be applied to common lactone and cyclic carbonate monomers. Further exploration of available PBGs, monomers, and crosslinkers, and expansion of the concept toward ring-opening copolymerization systems will open the door for sustainable 3DP technologies.
